# Low SARS-CoV-2 antibody titers may be associated with poor clinical outcomes for patients with severe COVID-19

**DOI:** 10.1038/s41598-022-12834-w

**Published:** 2022-06-01

**Authors:** Mumon Takita, Toru Yoshida, Tomoya Tsuchida, Yu Nakagama, Yasutoshi Kido, Shotaro Suzuki, Mitsuru Imamura, Kimito Kawahata, Goji Shimizu, Hideki Yoshida, Daiki Morikawa, Takeshi Kawaguchi, Shuichi Fujii, Jumpei Tsukuda, Takako Motohashi, Shigeki Fujitani

**Affiliations:** 1 Department of Emergency and Critical Care Medicine, St. Marianna University School of Medicine, Kawasaki, Kanagawa Prefecture 216‑8511 Japan; 2 Division of General Internal Medicine, Department of Internal Medicine, St. Marianna University School of Medicine, Kawasaki, Kanagawa Prefecture 216‑8511 Japan; 3 Department of Parasitology and Research Center for Infectious Disease Sciences, Graduate School of Medicine, Osaka City University, 1-4-3 Asahimachi Abeno-ku, Osaka, 545-8585 Japan; 4 Division of Rheumatology and Allergology, Department of Internal Medicine, St. Marianna University School of Medicine, Kawasaki, Kanagawa Prefecture 216-0015 Japan; 5 Department of Preventive Medicine, St Marianna University School of Medicine, Kawasaki, Kanagawa Prefecture 216‑8511 Japan

**Keywords:** SARS-CoV-2, Viral infection

## Abstract

Recently, immune response to coronavirus disease (COVID-19) has attracted attention where an association between higher antibody titer and worsening disease severity has been reported. However, our experiences with severe COVID-19 patients with low antibody titers led to hypothesizing that suppressed humoral immune response may be associated with poorer prognosis in severe COVID19. In this study, antibody titers in severe COVID19 patients were measured at 7, 10, 12, and 14 days after onset. Patients were divided into survivors and non-survivors. SARS-CoV-2 IgM in survivors and non-survivors were 0.06 AU and 0.02 AU (*P* = 0.048) at 10 days, 0.1 AU and 0.03 AU (*P* = 0.02) at 12 days, and 0.17 AU and 0.06 AU (*P* = 0.02) at 14 days. IgG in survivors and non-survivors were 0.01 AU and 0.01 AU (*P* = 0.04) at 7 days, 0.42 AU and 0.01 AU (*P* = 0.04) at 12 days, and 0.42 AU and 0.01 AU (*P* = 0.02) at 14 days. Multivariate analysis showed better survival among patients with IgM positivity at 12 days (*P* = 0.04), IgG positivity at 12 days (*P* = 0.04), IgM positivity at 14 days (*P* = 0.008), and IgG positivity at 14 days (*P* = 0.005). In severe COVID-19, low antibody titers on days 12 and 14 after onset were associated with poorer prognosis.

## Introduction

The first SARS-CoV-2 infections (coronavirus disease 2019, COVID-19) emerged in Wuhan, China, in December 2019 and had quickly spreaded around the world. The World Health Organization (WHO) statistics (up to April 25, 2021) reported 146,054,107 infections and 3,092,410 deaths worldwide^1^. The mortality rate associated with COVID-19 is reported to be approximately 3.5 times higher than that of seasonal influenza^2^. It is important to identify and respond early to severe COVID-19 cases as they may lead to death. Sex, history of hypertension and chronic renal failure, high C-reactive protein (CRP) levels, high D-dimer levels, and lymphocytopenia have been reported to be predictors of COVID-19 death^3^, and immune responses are considered to be related to severity. Humoral immunity to SARS-CoV-2 has been analyzed in depth^4^, and it is now well understood that immunoglobulin M (IgM) and immunoglobulin G (IgG) antibodies to the spike (S) and nucleocapsid (N) proteins of SARS-CoV-2 have been reported to both play essential yet mutually distinct roles in the host defense mechanism^5^. In severe acute respiratory syndrome (SARS), which is caused by an ancestral coronavirus of SARS-CoV-2, it has been reported that the sustainability of IgG production keeps eliminating the virus during recovery from the disease state^6^. A positive correlation between a high antibody titer and disease severity has also been reported^7^. In one study of Middle East Respiratory Syndrome (MERS), delay in antibody production response were related to severity, but the total number of patients included in that study was small^8^.

Several reports on COVID-19 and antibody titers have been published. Zhao et al. measured the antibody titer of 173 patients (141 non-critical and 32 critical) longitudinally during hospitalization and reported that the critical group had a higher antibody titer after the 12th day of onset, and the multivariate analysis showed that a high antibody titer contributed to severity^9^. In that study, patients with acute respiratory disease syndrome (ARDS) or patients with SpO_2_ < 93% who needed invasive or noninvasive respiratory support were defined as “critical”; however, the percentage of intubated patients was not reported^9^. Wang et al. investigated the dynamics of antibody responses in 23 patients with COVID-19 (12 severely ill and 11 mildly ill) and found that IgM response and neutralizing antibody titers were higher in the severely ill group^10^. All 12 patients with severe illness were under ventilator management. Liu et al. compared neutralizing antibody titers in 50 COVID-19 patients (eight intensive care unit [ICU] patients and 42 non-ICU patients) and reported that ICU patients had higher neutralizing antibody titers than those of non-ICU patients^11^. The S antigen-targeting antibody titer serves as the surrogate of viral neutralizability. Therefore the two indicators of humoral immunity, anti-S antibody titer and neutralizing antibody titer are positively correlated^12^, and cases with high anti-S antibody titers demonstrate sufficient viral growth suppression.

In previous reports^9–11^, COVID-19 antibody titers have been reported to be higher in critically ill patients. On the other hand, in our facility, we had patients with severe COVID-19 who had low antibody levels in response to SARS-CoV-2. Our initial experiences with a series of severely ill COVID-19 patients with persistently negative or very low antibody titers led to the hypothesis that a suppressed humoral immune response may be associated with worse prognosis in severely ill patients. This study was performed to verify whether a suppressed humoral immune response, assessed by measuring anti-S IgM and IgG, was associated with ICU mortality from severe COVID-19.

## Results

### Baseline patient characteristic

**Figure 1:**
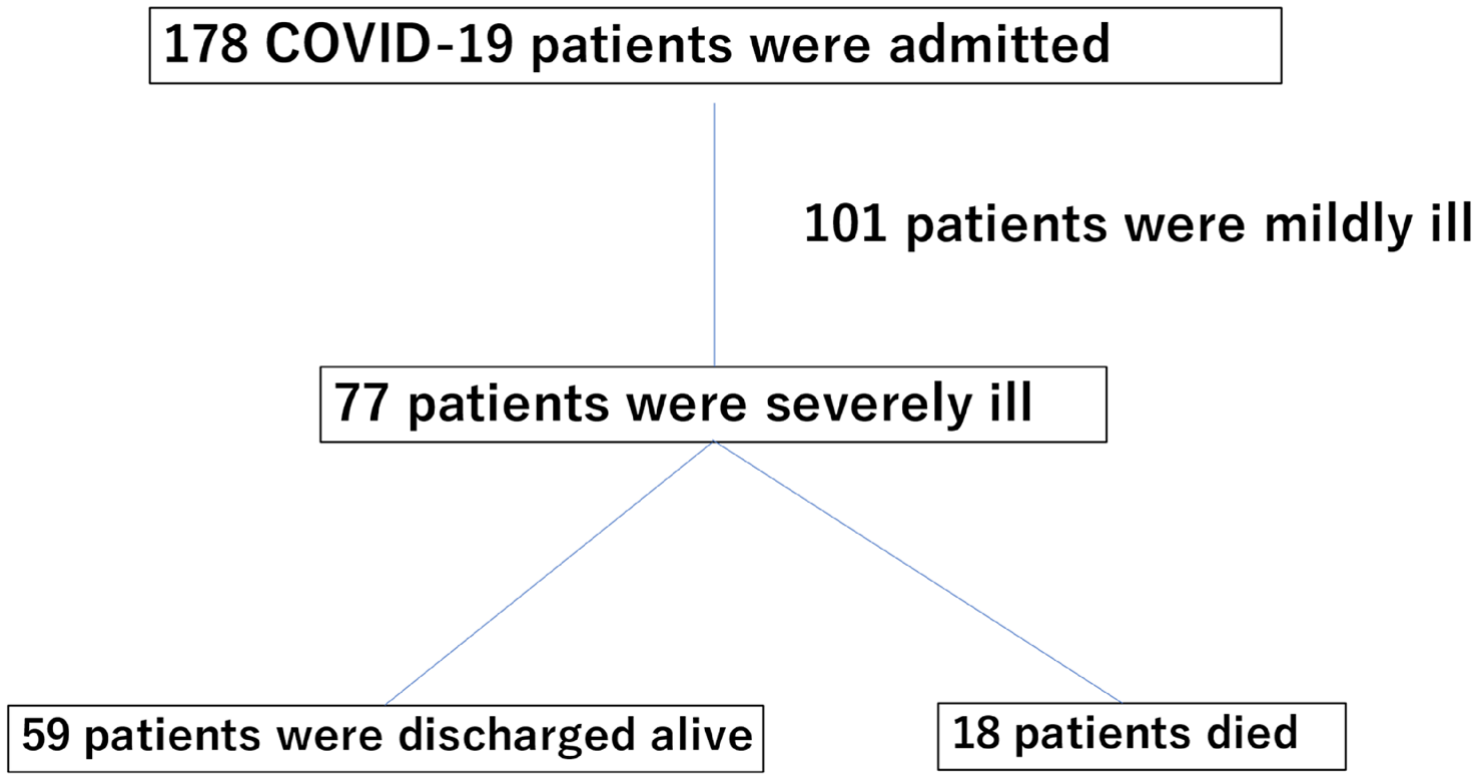
Flow chart of patient selection.

There were 59 survivors and 18 non-survivors (Fig. 1). Table 1 shows the background factors, underlying diseases, blood sampling findings at admission, and treatment details for the survivor and non-survivor groups. The median (IQR) age was 64.0 (55.0–74.0) years in the survivor group and 74.5 (65.0–84.3) years in the non-survivor group, and this difference was significant (*P* = 0.02). In terms of underlying diseases, eight (13.6%) patients in the survivor group and ten (55.6%) in the non-survivor group had chronic kidney failure, and this difference was significant (*P* < 0.05). The number of patients with permanent dialysis was three (5.10%) in the survivor group and eight (44.4%) in the non-survivor group, and this difference was significant (*P* < 0.05). Blood collection findings at hospitalization showed a lower lymphocyte count (7.90% [4.65–12.8] vs. 3.95% [2.15–7.03], *P* < 0.05) and a lower albumin level (2.90 g/dl [2.68–3.10] vs. 2.70 g/dl [2.28–2.93], *P* = 0.02) in the non-survivor group. CRP (8.31 mg/dl [4.85–13.4] vs. 14.7 mg/dl [6.13–21.9], *P* = 0.03), NT-proBNP (201 pg /ml [52.9–555] vs. 6584 pg/ml [731–20770], *P* < 0.05), procalcitonin (0.13 ng/ml [0.09–0.35] vs. 1.36 ng/ml [0.49–2.56], *P* < 0.05), BUN (21.5 mg/dl [15.4–42.8] vs. 50.0 mg/dl [29.6–74.4], *P* < 0.05), and Creatinine levels (0.87 mg/dl [0.63–1.28] vs. 3.92 mg/dl [0.94–9.02], *P* < 0.05) were significantly higher in the non-surviving group. In addition, the PaO_2_/FiO_2_ ratio was significantly lower in the non-survivor group than in the survivor group (220 [150–265] vs. 165 [121–227], *P* = 0.04). The standard patient treatment regimens included antimicrobial drugs (azithromycin and ceftriaxone for community-acquired pneumonia), steroids (dexamethasone 6 mg/day), and remdesivir. Remdesivir was not administered to patients with chronic renal failure.

**Table 1:** Patient characteristics.

		Total (n = 77)	Survivor (n = 59)	Non-survivor (n = 18)	*P* value
Age, years	Median [IQR]	68.0 [57.0–77.0]	64.0 [55.0–74.0]	74.5 [65.0–84. 3]	0.02
Sex (male %)	n (%)	64.0 (83.0)	50.0 (84.0)	14.0 (78.0)	0.49
**Past medical history**					
Hypertension	n (%)	48.0 (62.3)	36.0 (61.0)	12.0 (66.7)	0.19
COPD	n (%)	6.00 (7.80)	3.00 (5.10)	3.00 (16.7)	0.14
BA	n (%)	8.00 (10.4)	6.00 (10.2)	2.00 (11.1)	1.00
Chronic lung disease	n (%)	6.00 (7.90)	3.00 (5.20)	3.00 (16.7)	0.14
Chronic kidney disease	n (%)	18.0 (23.4)	8.00 (13.6)	10.0 (55.6)	< 0.05
Permanent dialysis	n (%)	11.0 (14.3)	3.00 (5.10)	8.00 (44.4)	< 0.05
CHF	n (%)	5.00 (6.50)	4.00 (6.80)	1.00 (5.60)	1.00
NYHA I·II		5.00 (100)	4.00 (80.0)	1.00 (20.0)	1.00
NYHA III·IV		0.00 (0.00)			
Ischemic heart disease	n (%)	7.00 (9.20)	3.00 (5.20)	4.00 (22.2)	0.05
Cerebrovascular disease	n (%)	11.0 (14.3)	7.00 (11.9)	4.00 (22.2)	0.27
Diabetes mellitus	n (%)	31.0 (40.3)	23.0 (39.0)	8.00 (44.4)	0.79
Insulin user		9.00 (29.0)	7.00 (30.4)	2.00 (25.0)	1.00
Hyperuricemia	n (%)	10.0 (13.0)	8.00 (13.6)	2.00 (11.1)	1.00
Hyperlipemia	n (%)	23.0 (29.9)	19.0 (32.2)	4.00 (22.2)	0.56
BMI	Median [IQR]	25.2 [22.9–29.8]	25.9 [23.4–30.4]	24.4 [20.8–28.0]	0.16
Obesity (BMI ≥ 30)	n (%)	15.0 (19. 5)	14.0 (25.9)	1.00 (5.56)	0.09
History of smoking	n (%)	42.0 (54. 6)	33.0 (67.4)	9.00 (56.3)	0.37
Past smoker		32.0 (76.2)	24.0 (52.2)	8.00 (50.0)	1.00
Smoking		10.0 (23.8)	9.00 (21.4)	1.00 (7.69)	0.42
Malignant tumor	n (%)	12.0 (15.6)	8.00 (13.6)	4.00 (22.2)	0.46
Active	n (%)	3.00 (25.0)	1.00 (12.5)	2.00 (50.0)	0.24
Not active	n (%)	9.00 (75.0)	7.00 (87.5)	2.00 (50.0)	0.24
Immunodeficiency after solid organ transplantation	n (%)	1.00 (1.30)	1.00 (1.70)	0.00 (0.00)	1.00
Steroid user	n (%)	2.00 (2.60)	2.00 (3.40)	0.00 (0.00)	1.00
> 10 mg/day (over 1 month)		1.00 (50.0)	1.00 (50.0)	0.00 (0.00)	1.00
Immunosuppressant user	n (%)	4.00 (5.40)	3.00 (5.40)	1.00 (5.60)	1.00
Biological product users	n (%)	1.00 (1.30)	1.00 (1.70)	0.00 (0.00)	1.00
**Laboratory findings (at admission)**					
WBC (10^3^/μL)	Median [IQR]	7.50 [5.30–11.0]	7.10 [5.00–9.60]	8.95 [6.05–13.8]	0.10
Lymphocytes (%)	Median [IQR]	6.70 [4.00–10.5]	7.90 [4.65–12.8]	3.95 [2.15–7.03]	< 0.05
Neutrophil (%)	Median [IQR]	86.4 [82.9–91.0]	86.4 [79.3–91.5]	88.6 [85.2–90.6]	0.26
CD3 +/CD4 + (%)	Median [IQR]	34.2 [26.1–42.9]	34.2 [26.9–42.0]	31.2 [24.3–46.7]	0.96
CD3 +/CD8 + (%)	Median [IQR]	13.6 [8.90–19.3]	13.2 [8.90–19. 2]	14.1 [11.1–28.6]	0.37
CD4/CD8	Median [IQR]	2.45 [1.54–3.66]	2.42 [1.72–3.68]	2.61 [0.85–3.66]	0.55
CRP (mg/dl)	Median [IQR]	9.02 [4.96–16.2]	8.31 [4.85–13.4]	14.7 [6.13–21.9]	0.03
Alb (g/dl)	Median [IQR]	2.90 [2.60–3.10]	2.90 [2.68–3.10]	2.70 [2.28–2.93]	0.02
PT-INR	Median [IQR]	1.12 [1.06–1.19]	1.11 [1.05–1.17]	1.16 [1.10–1.22]	0.05
D-dimer (μg/ml)	Median [IQR]	1.60 [1.00–5.30]	1.40 [0.80–4.95]	2.80 [1.43–9.05]	0.08
Fibrinogen (mg/dl)	Median [IQR]	570 [470–643]	570 [461–660]	551 [478–631]	0.88
LDH (U/L)	Median [IQR]	440 [363–605]	431 [362–616]	524 [365–595]	0.66
CK (U/L)	Median [IQR]	85.0 [51.0–229]	83.0 [50.0–210]	105 [56.3–295]	0.39
NT-proBNP (pg/ml)	Median [IQR]	334 [85.9–1422]	201 [52.9–555]	6584 [731–20770]	< 0.05
AST (U/L)	Median [IQR]	43.0 [30.5–57.0]	43.0 [33.0–59.0]	36.5 [21.3–56. 3]	0.28
ALT (U/L)	Median [IQR]	30.0 [19.0–44.5]	32.0 [21.0–45.0]	20.0 [12.0–42. 3]	0.06
Ferritin (ng/ml)	Median [IQR]	812 [462–1274]	818 [488–1270]	757 [291–1495]	0.53
Procalcitonin (ng/ml)	Median [IQR]	0.19 [0.09–0.72]	0.13 [0.09–0.35]	1.36 [0.49–2.56]	< 0.05
BUN (mg/dl)	Median [IQR]	26.7 [17.5–49.0]	21.5 [15.4–42.8]	50.0 [29.6–74.4]	< 0.05
Cre (mg/dl)	Median [IQR]	0.97 [0.69–2.15]	0.87 [0.63–1.28]	3.92 [0.94–9.02]	< 0.05
KL-6 (U/ml)	Median [IQR]	341 [260–647]	322 [255–556]	595 [294–1112]	0.07
Pulmonary surfactant A (ng/ml)	Median [IQR]	63.1 [37.7–92.5]	61.2 [36.5–81.8]	75.2 [47.1–120]	0.29
Pulmonary surfactant D (ng/ml)	Median [IQR]	87.9 [36.1–194]	77.4 [34.7–193]	97.7 [56.3–258]	0.39
P/F ratio on admission	Median [IQR]	205 [142–255]	220 [150–265]	165 [121–227]	0.04
**Treatment medication**					
Dexamethasone	n (%)	77 (100)	59 (100)	18 (100)	–
Remdesivir	n (%)	65 (84.4)	51 (86.4)	14 (77.8)	0.46
Tocilizumab	n (%)	5 (6.5)	4 (6.78)	1 (5.56)	1.00
Heparin	n (%)	73 (94.8)	57 (96.6)	16 (88.9)	0.23
**Respiratory therapy**					
Oxygen therapy	n (%)	8.00 (10.4)	8.00 (13.6)	0.00 (0.00)	
Nasal high flow	n (%)	28.0 (36.4)	21.0 (35. 6)	7.00 (38. 9)	0.80
Ventilator	n (%)	42.0 (54.6)	31.0 (52.5)	11.0 (61.1)	0.52
Duration of ventilator	Median [IQR]	14.0 [13.0–21.3]			
ECMO	n (%)	3.00 (3.90)	2.00 (3.39)	1.00 (5.56)	0.56

*P* < 0.05 indicates a significant difference.

*COPD* chronic obstructive pulmonary disease, *BA* Bronchial asthma, *CHF* congestive heart failure, *NYHA* New York Heart Association, *BMI* body mass index, *WBC* white blood cell, *IQR* interquartile range, *CRP* C-reactive protein, *Alb* albumin, *PT* prothrombin time, *LDH* lactate dehydrogenase, *Cre* creatinine, *CK* creatinine kinase, *NT-proBNP* N‑terminal pro‑B‑type natriuretic peptide, *AST* aspartate amino transferase, *ALT* alanine amino transferase, *BUN* blood urea nitrogen, *KL-6* Krebs von den Lungen-6, *P/F ratio* partial arterial oxygen pressure/fraction of inspiration oxygen ratio, *ECMO* extracorporeal membranous oxygenation.

### Antibody titers

**Figure 2:**
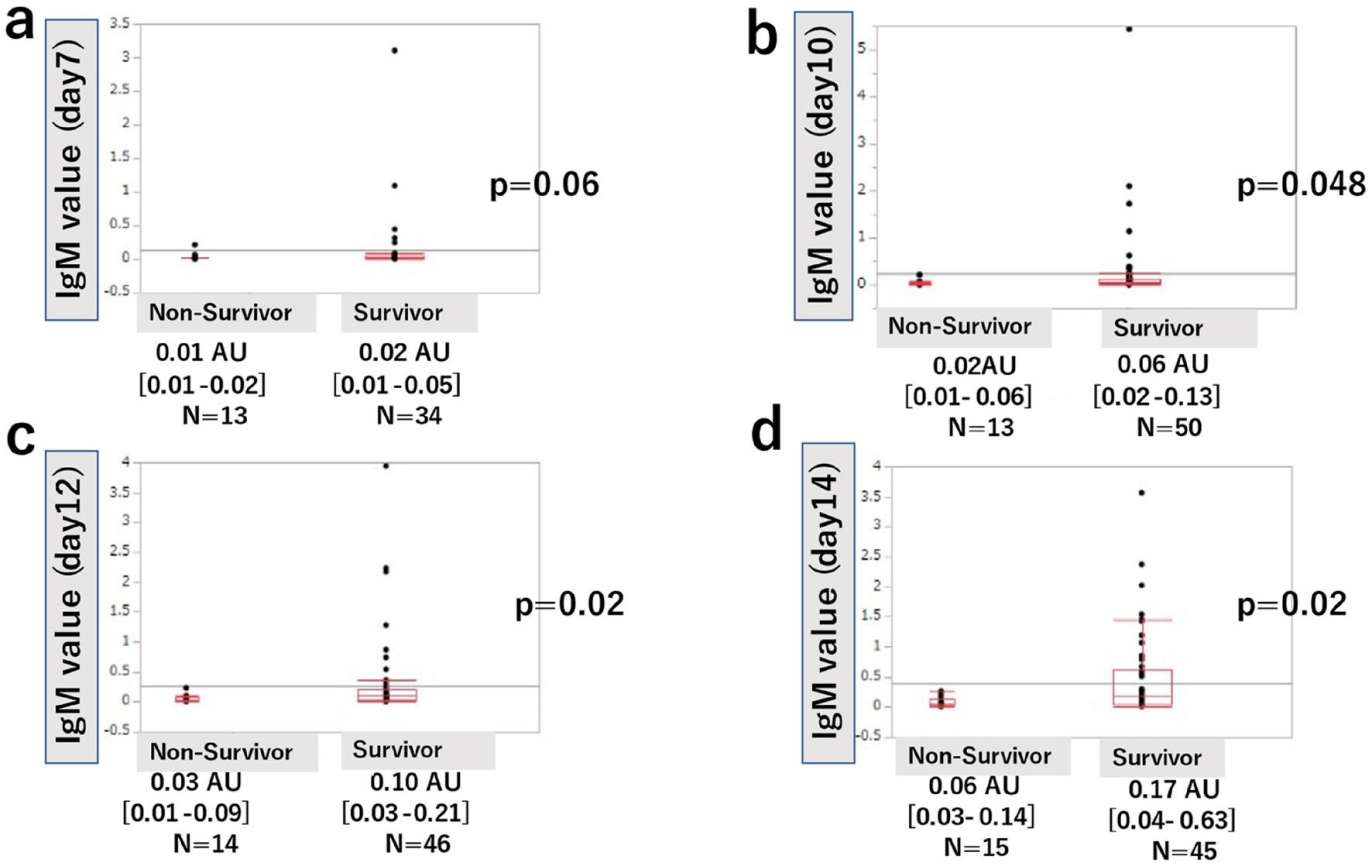
Comparison of daily IgM values between survivors and non-survivors. Antibody titers of IgM on day 7 after onset were not significantly different between the survivor and non-survivor groups (**a**). Antibody titers of IgM on day 10, 12, and 14 after onset were significantly higher in the survivor group (**b**, **c**, **d**). Values represent median and interquartile range. *N* total number of patients. *P* < 0.05 indicates a significant difference. *IgM* immunoglobulin M.

**Figure 3:**
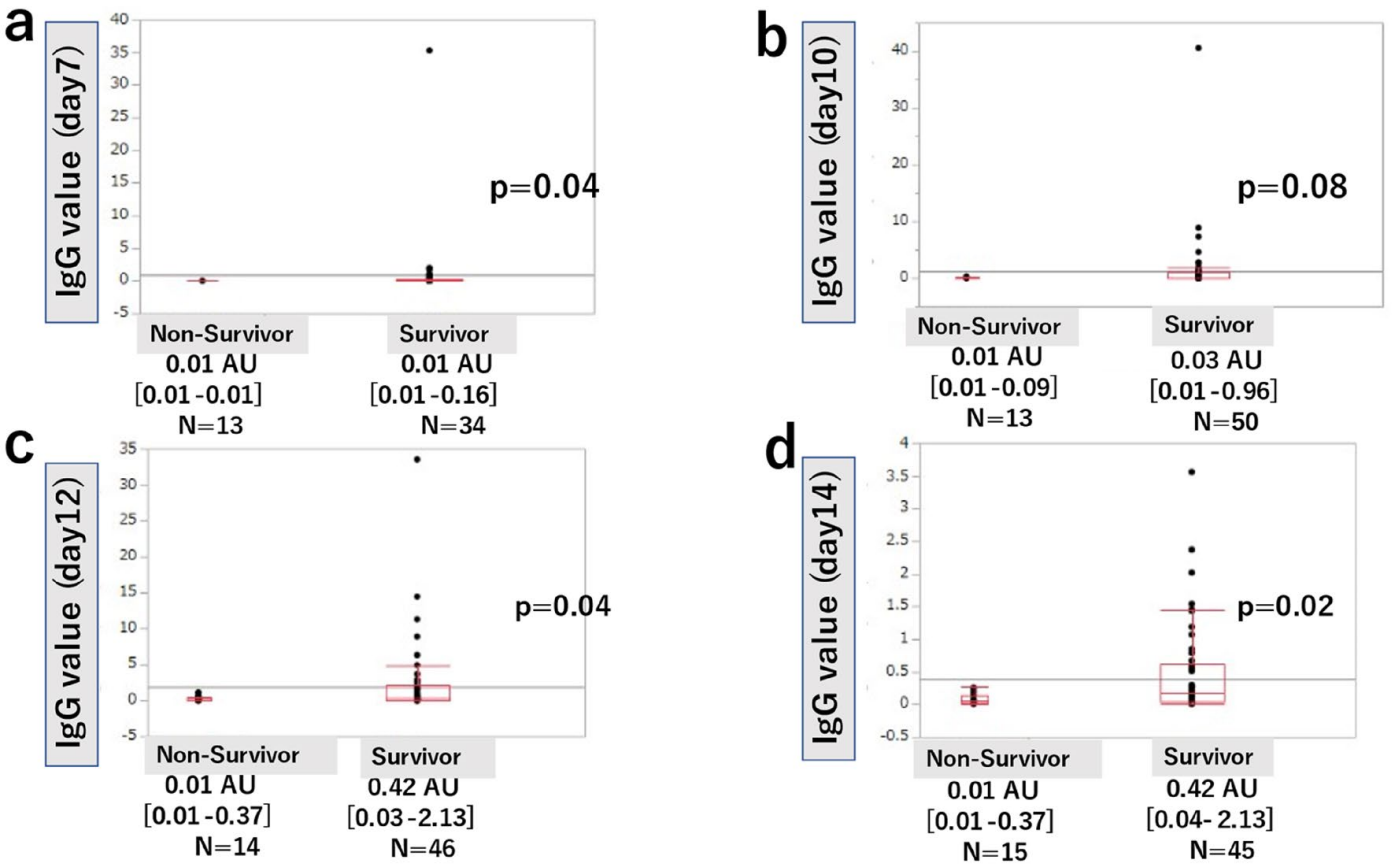
Comparison of daily IgG values between survivors and non-survivors. Antibody titers of IgG on day 10 after onset were not significantly different between the survivor and non-survivor groups (**b**). Antibody titers of IgG on day 7, 12, and 14 after onset were significantly higher in the survivor group (**a**, **c**, **d**). Values represent median and interquartile range. *N* total number of patients. *P* < 0.05 indicates a significant difference. *IgG* immunoglobulin G.

Figures 2 and 3 show a comparison of the median daily antibody titers between the survivor and non-survivor group. For IgM at 7 days after onset, there was no significant difference between the survivor group and the non-survivor group (0.02 AU [0.01–0.05] vs. 0.01 AU [0.01–0.02], *P* = 0.06), but there was a significant difference between the survivor group and the non-survivor group for IgM at 10 days, 12 days, and 14 days (0.06 AU [0.02–0.13] vs. 0.02 AU [0.01–0.06], *P* = 0.048, 0.10 AU [0.03–0.21] vs. 0.03 AU [0.01–0.09], *P* = 0.02, and 0.17 AU [0.04–0.63] vs. 0.06 AU [0.03–0.14], *P* = 0.02, respectively). For IgG at 7 days after onset, there was a significant difference between the survivor group and the non-survivor group (0.01 AU [0.01–0.16] vs. 0.01 AU [0.01–0.01], *P* = 0.04), but there was no significant difference between the survivor group and the non-survivor group for IgG at 10 days (0.03 AU [0.01–0.96] vs. 0.01AU [0.01–0.09], *P* = 0.08). For IgG at 12 and 14 days after onset, there were significant differences between the survivor group and the non-survivor group (0.42 AU [0.03–2.13] vs. 0.01 AU [0.01–0.37], *P* = 0.04 and 0.42 AU [0.04–2.13] vs. 0.01 AU [0.01–0.37], *P* = 0.02, respectively).

### Cutoff value calculated from ROC curve

We created a ROC curve for each day’s antibody value with survival as an outcome. IgM at 10, 12, and 14 days after onset and IgG at 7, 12, and 14 days after onset were significantly different in survivors and non-survivors. Cutoff values were established based on these values (Table 2, Supplementary Figs. S1–S4 online). When the antibody level was above the cutoff, it was categorized as positive, and when the antibody level was less than the cutoff, we categorized it as negative. Based on these cutoffs, the IgM positive rate at 10 days after onset and the IgM and IgG positivity rate at 12 and 14 days after onset were higher in the survivor group than in the non-survivor group (Table 3).

**Table 2:** Cutoff values of the antibody levels.

	Cutoff (obtained from ROC curve, refer to Supplementary Figs. S3–S6 online)
IgG (day 7)	0.02 (Sensitivity 38.4%, Specificity 92.3%, AUC 0.66)
IgM (day 10)	0.08 (Sensitivity 40.0%, Specificity 92.3%, AUC 0.68)
IgM (day 12)	0.11 (Sensitivity 47.8%, Specificity 92.3%, AUC 0.70)
IgG (day 12)	0.58 (Sensitivity 45.7%, Specificity 92.9%, AUC 0.68)
IgM (day 14)	0.27 (Sensitivity 42.2%, Specificity 100%, AUC 0.70)
IgG (day 14)	1.65 (Sensitivity 55.6%, Specificity 86.7%, AUC 0.69)

*Ig* immunoglobulin, *ROC* receiver operating characteristic, *AUC* area under the concentration–time curve.

**Table 3:** Daily rates of IgM and IgG positivity (based on cutoff values).

		Survivor n (%)	Non-survivor n (%)	*P* value
Day 7	IgM-positive	–	–	–
	IgG-positive	13 (38.24)	1 (7.69)	0.07
Day 10	IgM-positive	20 (40.00)	1 (7.69)	0.04
	IgG-positive	–	–	–
Day 12	IgM-positive	22 (47.83)	1 (7.14)	0.01
	IgG-positive	21 (45.65)	1 (7.14)	0.01
Day 14	IgM-positive	18 (40.00)	0 (0.00)	0.03
	IgG-positive	25 (55.56)	2 (13.33)	0.01

IgG positivity (day 7) is defined as IgG (day 7) ≥ 0.02 (0.02 is obtained from the ROC curve).

IgM positivity (day 10) is defined as IgM (day 10) ≥ 0.08 (0.02 is obtained from the ROC curve).

IgM positivity (day 12) is defined as IgM (day 12) ≥ 0.11 (0.11 is obtained from the ROC curve).

IgG positivity (day 12) is defined as IgG (day 12) ≥ 0.58 (0.58 is obtained from the ROC curve).

IgM positivity (day 14) is defined as IgM (day 14) ≥ 0.27 (0.27 is obtained from the ROC curve).

IgG positivity (day 14) is defined as IgG (day 14) ≥ 1.65 (1.65 is obtained from the ROC curve).

There are no differences in IgM (day 7) and IgG (day 10) between survivors and non-survivors. Therefore, the cutoff values for IgM (day 7) or IgG (day 10) are not set, and positivity rates are not set.

Fisher’s test is conducted if sample numbers are less than 5.

*P* < 0.05 indicates statistical significance.

*Ig* immunoglobulin, *ROC* receiver operating curve.

### Kaplan–Meier curve

**Figure 4:**
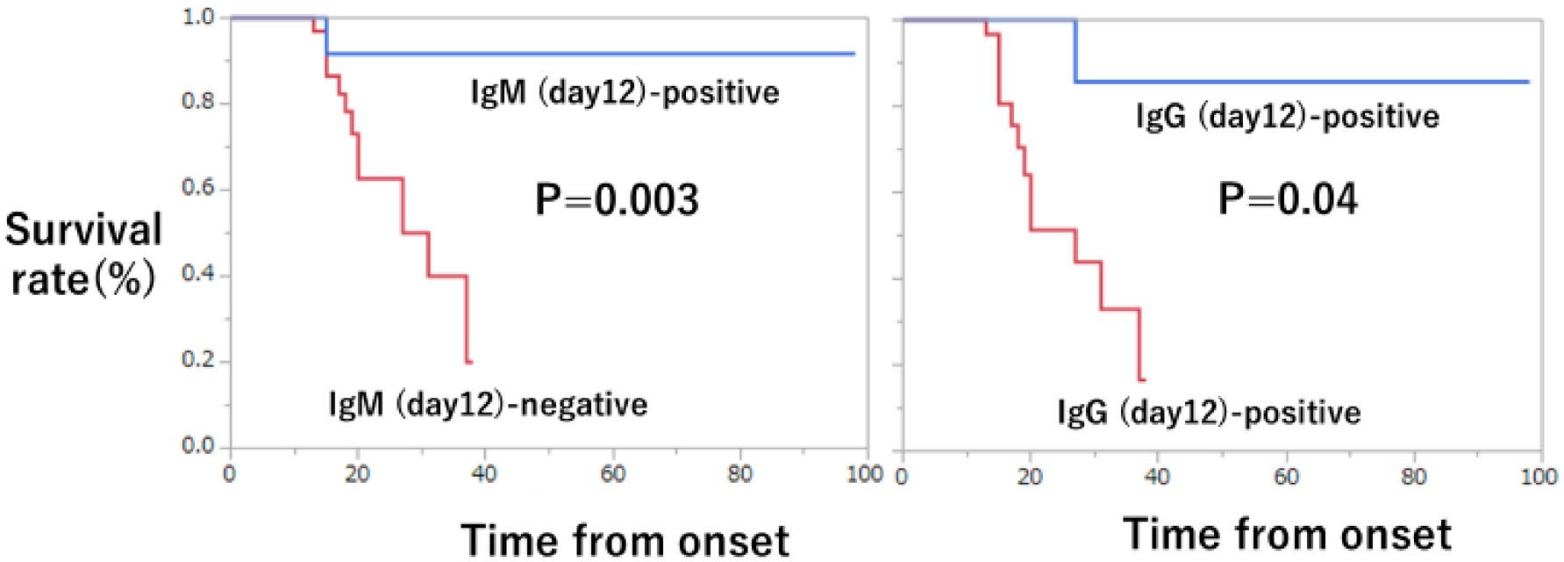
Kaplan–Meier curve on day 12. Kaplan–Meier curves were drawn for antibody-positive and negative cases 12 days after onset with survival as the outcome. IgM/IgG measured values above the cutoff values in Table 2 (IgM ≥ 0.11, IgG ≥ 0.58) were defined as antibody-positive, and those below the cutoff values were defined as antibody-negative. For both, IgM and IgG, the antibody-positive group showed significant differences in survival.

Table 3 demonstrates the days when there was a significant difference in the antibody positivity rate between the two groups (IgM: days 10, 12, and 14; IgG: days 12 and 14). Kaplan–Meier curves were drawn with survival as the outcome (Supplementary Figs. S5 and S6, Fig. 4). No significant difference was observed on day 10, but significant differences were observed after day 12.

### Multivariate analysis with survival as the outcome

Table 4 shows the results of four multivariate analyses with survival as the outcome. First, we adopted IgM positivity (IgM ≥ 0.11 AU) 12 days after onset, and the levels of CRP and lymphocytes upon admission as the modulating factors. Based on the results, we inferred that IgM positivity on day 12 could be an independent predictor of survival (odds ratio [OR]: 94.9, CI: 3.54–2550, *P* = 0.01). Second, we adopted IgG positivity (IgG ≥ 0.58 AU) 12 days after onset, age, and maintenance dialysis as the modulating factors. Results showed that IgG positivity on day 12 could be an independent predictor of survival (OR: 7.60, CI: 1.31–153, *P* = 0.04). Third, we adopted IgM positivity (IgM ≥ 0.27 AU) 14 days after onset, maintenance dialysis, and CRP upon admission as the modulating factors. IgM positivity on Day 14 was also an independent predictor of survival (OR: 1.75 × 10^9^, CI: 7.36–, *P* < 0.01). Finally, when we adopted IgG positivity (IgG ≥ 1.65 AU) 14 days after onset, age, and maintenance dialysis as the modulating factors, IgG positivity on Day 14 was found to be an independent predictor of survival (OR: 11.5, CI: 1.94–127, *P* = 0.01).

**Table 4:** Multivariate logistic regression analysis for survival.

	Odds ratio	95% confidence interval	*P* value
**IgM (day 12) ≥ 0.11**	94.9	3.54–2550	0.01
CRP at admission	0.82	0.71–0.94	< 0.01
Lymphocytes at admission	1.00	0.99–1.01	0.08
**IgG (day 12) ≥ 0.58**	7.60	1.31–153	0.04
Age	0.93	0.89–0.99	0.02
Maintenance dialysis	0.04	0.002–0.28	< 0.01
**IgM (day 14) ≥ 0.27**	1.75 × 10^9^	7.36-	< 0.01
Maintenance dialysis	0.04	0.002–0.35	< 0.01
CRP at admission	0.85	0.74–0.96	0.01
**IgG (day 14) ≥ 1.65**	11.5	1.94–127	0.01
Age	0.89	0.80–0.96	< 0.01
Maintenance dialysis	0.01	0.0002–0.12	< 0.01

*P/F* PaO_2_/FiO_2_, *Ig* immunoglobulin, *CRP* C-reactive protein.

### Comparison with other antibody kits

The antibody kit used in this study was found to correlate with the Architect SARS-CoV-2 IgM antibody measurement kit (Abbott) and the Architect SARS-CoV-2 IgG II Quant (Abbott) (Supplementary Table S7 online).

## Discussion

In our study, the antibody titers were significantly higher in the survival group, and multivariate analysis showed that higher antibody titers (IgM and IgG) at 12 and 14 days after the onset of COVID-19 were associated with increased chance of survival.

Previous studies have reported that antibody titers were higher in severe COVID-19 than those in moderate COVID-19^9–11,13^. To the best of our knowledge, there are no reports of antibody measurements in a population focusing on severe COVID-19 cases. One report compared the antibody positivity rates of a moderate group, a severe group, and a critical group, and no significant difference was found between them^14^. However, in that study, antibody levels were measured at 14 days or more from the onset of COVID-19 in more than 80% of cases, and were not measured at the earlier stages of 10–14 days after onset. It has been reported that the condition of COVID-19 patients worsens during the period 10–14 days after onset^13^; therefore, mortality was not related with antibody titer in that previous study^14^. In our study, the assessment of antibody titers from the earlier stage of illness at 10–14 days after onset allowed us to demonstrate the prognostic significance of antibody titers in predicting mortality. The antibody titer shows promise as a prognostic predictor in patients with severe COVID-19 since antibody titers greater than the set cutoff values at 12 and 14 days contributed to a better chance of survival in our multivariate analysis. It has been reported that the intensity of acquired immune response during the course of sepsis or influenza pneumonia is associated with prognosis^15–17^. In our patient cohort, using the cutoff values shown in Table 2, the antibody titers could predict survival with high specificity. Patients with a higher antibody titer on day 12 or 14 were more likely to survive, regardless of similarity in clinical severity.

Convalescent plasma therapy, while still investigational, has been reported to lower mortality rates when plasma with high neutralizing antibody titers are administered early after diagnosis (within 3 days)^18,19^. A positive effect is expected if especially the patients receiving convalescent plasma therapy are severely ill COVID-19 patients with low antibody titers in the early stage. Our observation led to a striking speculation that the significant portion of ICU-admitted severely ill COVID-19 patients exhibiting a suppressed humoral immune response and thus following a poor prognosis, may benefit from immune augmentation with passive immunoglobulin therapy. Moreover, convalescent plasma therapy may even be considered for patients with severe COVID19 with low antibody titers within 14 days of onset. However, the timing of antibody titer measurement was at 7, 10, 12, and 14 days from onset in our study, and as this was more than 3 days after onset (the suggested timing in the previous study for plasma infusion)^19^, the question remains to be solved on whether the group of patients with low antibody titer in our study would have benefited from convalescent plasma therapy.

The mortality rate in our study group was 23.4%, higher than that reported in the three previously published studies assessing antibody titers, 0%^10^ 1.1%^9^, and 18%^11^, respectively. On the other hand, in a systematic review^20^ of patients with severe COVID-19 who entered the ICU or high dependency unit, the average age was 62.6 years and the hospital mortality rate was 28.1% (95% CI 23.4–33.0), which is similar to our report. In treatment, 67.7% (95% CI 59.1–75.7) of patients needed a ventilator and 6.4% (95% CI 4.1–9.1) needed ECMO was similar to the treatment received by the patients in our study^20^. Moreover, when we compared the survivor group and the non-survivor group in our study, age was significantly higher in the non-survivor group, and this is consistent with past reports^21,22^. In terms of underlying diseases, about half of the non-survivor group were receiving permanent dialysis, and the proportion of permanent dialysis patients was significantly higher in the non-survivor group. In a retrospective cohort study conducted at 68 US facilities, 143 of the 4,264 severe COVID-19 patients admitted to the ICU were permanent dialysis patients, and dialysis patients had a higher mortality rate in the hospital than those without chronic renal failure (adjusted HR, 1.41 [95% CI, 1.09–1.81])^23^. The results of this study^23^ were similar to that of our study.

Blood test results at admission showed a significant decrease in lymphocyte count, and a significant elevation in CRP, NT-proBNP, and procalcitonin levels in the non-survivor group. Our findings were similar to the findings from a systematic review reporting lymphocyte count, CRP, NT-proBNP, and procalcitonin levels as risk factors of mortality^3^. The P/F ratio at admission was significantly lower in the non-survivor group in our study, which is also consistent with previous reports^21^.

Vaccination status and SARS-CoV-2 variants are potential influential factors on mortality^24,25^. Vaccinations in Japan, however, started on February 17, 2021^26^, and therefore, could not have affected the interpretation of the results of our study, which was conducted between August 1, 2020, and February 9, 2021. Also, variant infections were not prevalent during our study period (August 1, 2020, to February 9, 2021)^27^, and therefore, we assumed there was little effect on our study.

The strength of our study was that we compared the COVID-19 antibody titers in a population of severe patients, and we showed a relationship between antibody titer and prognosis. In addition, antibody titers were measured and compared at 7–14 days when the patient’s condition had worsened. As we performed a multivariate analysis to demonstrate the relationship between antibody titer and prognosis, we believe that the measurement of antibody titer can help in the management of critically ill COVID-19 patients.

However, the following limitations required consideration. First, this was a single-facility, prospective, cohort study. Second, the number of included patients was small. Specifically, only 18 patients were included in the non-survivor group, so the number of regulators in the multivariate analysis was limited. In Table 1, 10 factors demonstrated significant differences between survivors and non-survivors; however, we had to limit the number of factors analyzed to two or three due to the small cohort. Similarly, evaluating other factors in the multivariate analysis that might affect survival as regulators, including those that may not differ significantly, would be ideal. However, we could only extract the regulatory factors that seemed to have a high impact on survival for multivariate analysis. Third, the antibody-positive/negative cutoff values obtained in this study were targeted at specific populations (severely ill patients) and may be limited in terms of generalization to patients who do not have a severe degree of COVID-19. Fourth, although days after onset was used to set the antibody measurement points, information regarding days was obtained from patients, and therefore, there is a possibility of errors. Fifth, there were cases where antibody titers could not be measured for all of the days because a long time had elapsed between onset and hospitalization (for example, in cases where the time between onset and hospitalization was 13 days, antibody titers could only be measured on day 14). Sixth, the patients were followed up until the time of discharge. Our study included patients who were transferred to another medical institution after discharge, and the prognosis after discharge was not investigated. Seventh, the neutralizing antibody titer was not measured, and it was not confirmed that the obtained antibody titer was sufficient to neutralize the virus. However, antibody titers and neutralizing antibody titers are reported to be correlated^7^. Finally, the cutoff values for antibodies derived from the cohort in this study were applied to the same cohort, resulting in a certain degree of circular logic. External validation in other cohorts is warranted.

In conclusion, in patients with severe COVID-19, there was a higher antibody titer in the survivor group than that in the non-survivor group, and having a sufficient antibody titer on the 12th and 14th days after onset may be associated with better prognosis.

## Methods

### Purpose of research

In patients with severe COVID-19, we compared antibodies in the survivor and non-survivor groups. We then evaluated whether the period from onset to antibody positivity could be a prognostic predictor.

### Patients

This was a single-facility, prospective observational study. We studied severe COVID-19 patients admitted to the Critical Care Center at St. Marianna University School of Medicine Hospital between August 1, 2020 and February 9, 2021. The definition of severe disease was “patients who needed ventilators (including extracorporeal membrane oxygenation: ECMO) or patients who needed high concentration oxygen therapy of 5 L or more.” The definition of illness severity varied across guidelines. Japanese guidelines defined severe illness in patients who required ventilator management or intensive care management^28^. This study was conducted during the COVID-19 pandemic, and each municipality made its own efforts to avoid medical disruption. In the municipality where this study was conducted, secondary care centers that treated moderately ill patients and tertiary care centers that treated severely ill patients, such as our hospital, were separated to improve the efficiency of medical care. The severity of illness adopted in this study was based on the criteria of transporting patients from secondary to tertiary care centers, as determined by the municipality. Those who had already been infected or had close contact with COVID-19 patients more than a month ago were excluded. In the study population, we collected the date of COVID-19 onset, medical history, blood collection data at hospitalization, time series of antibody titer during hospitalization, and clinical information, and compared these factors in a survival discharge group (survivor group) and a death discharge group (non-survivor group). The onset date was the date when symptoms (fever, cough, etc.) related to COVID-19 appeared. For patients without ventilators and nasal high flow (NHF), FiO_2_ was set at the inhaled oxygen flow rate (oxygen 5 L /FiO_2_ 0.4, oxygen 6 L/FiO_2_ 0.5, oxygen 7 L/FiO_2_ 0.6, oxygen 8 L/FiO_2_ 0.8, oxygen 9 L/FiO_2_ 0.9).

### Antibody measurement

The antibody titer of patients was measured at 7 days, 10 days, 12 days, and 14 days after onset. For antibody measurement, a SARS-CoV-2 IgM and IgG Quantum Dot Immunoassay (Mokobio Biotechnology R&D Center, Rockville, MD, USA) was used. Later, we compared antibody titers between the survival group and the non-survival group. Although there are differences in the literature^9,29,30^, it has been reported that the time of antibody expression is 10–14 days from the onset. On the other hand, it has been reported that the condition of COVID-19 patients began to progressed in severity 10–14 days after the onset of symptoms^13^. Therefore, we measured antibody titers on day 7 through day 14 after onset.

### Antibody measurement kit

We used a SARS-CoV-2 IgM and IgG Quantum Dot Immunoassay as an antibody measurement kit. The kit detects IgM and IgG specific to SARS-CoV-2 S antigen using an immunochromatographic method^31^. The kit's sensitivity and specificity are reported to be 100% and 72.5%, respectively^32^. Antibody titers measured with the Quantum Dot Immunoassay were tested for correlation with chemiluminescent immunoassays: Architect SARS-CoV-2 IgM and Architect SARS-CoV-2 IgG II Quantitative antibody measurement kits (Abbott, Illinois, USA)^33^.

### Statistical analyses

The median (interquartile range) was used to represent continuous variables. For univariate analysis, the Wilcoxon test was used to compare continuous variables between the two groups, and the chi-square test was used to compare categorical variables. In the comparisons of categorical variables, Fisher’s test was used if the sample counts were less than five. When the antibody titer was significantly different between survivors and non-survivors in the univariate analysis on each day, we created a receiver operating characteristic (ROC) curve with survival as an outcome and with set cutoff values. On each day, we calculated the antibody positivity rate; when antibody value was more than cutoff, this was considered antibody-positive in the survival and non-survival groups. We then compared the positive rate in these groups. Kaplan–Meier curves were drawn with survival as the outcome at the date when the antibody-positive rate was significantly different between the two groups. On days when there was a significant difference in the survival curves, we performed a multivariate analysis to identify antibody positivity associated with survival. In the multivariate analysis, we considered factors that showed significant differences in the univariate analysis including background information, as well as age, sex, and underlying diseases (i.e., chronic obstructive pulmonary disease, type 2 diabetes, hypertension, dyslipidemia, and hyperuricemia), which were associated with risk factors for severity^21,22^. Finally, we selected seven regulatory factors: age, maintenance dialysis, P/F ratio at admission, lymphocyte count at admission, CRP at admission, procalcitonin, and Alb. However, due to the limited number of patients, we could only select two factors for analysis. Therefore, we created 21 models by pairing two regulators from each of these seven items and performed multivariate analysis for each model. As a result, the model with the best fit was adopted (Supplementary Tables S8–S11 online). Spearman's rank correlation coefficient was used as the correlation coefficient. Statistical significance was set at *P* < 0.05. Statistical analyses were performed using JMPR 13.0.0 (SAS Institute Inc., Cary, NC, USA).

### Ethics approval and consent to participate

This study was approved by the Life Ethics Committee of St. Marianna University School of Medicine (authorization number: 4940) and the Ethical Committee of Osaka City University Graduate School of Medicine (authorization number: 2020-003). Confirms that informed consent was obtained from all participants and/or their legal guardians. All methods were performed in accordance with relevant guidelines and regulations.

## Supplementary Information

Supplementary Information.

## Data Availability

The datasets used and analyzed during the current study are available from the corresponding author upon reasonable request.

## References

[1] World Health Organization. *Coronavirus Disease (COVID19) Pandemic*. https://www.who.int/emergencies/diseases/novel-coronavirus-2019. (Accessed 26 April 2021).

[2] Xie Y, Bowe B, Maddukuri G, Al-Aly Z (2020). Comparative evaluation of clinical manifestations and risk of death in patients admitted to hospital with covid-19 and seasonal influenza: Cohort study. BMJ.

[3] Tian W (2020). Predictors of mortality in hospitalized COVID-19 patients: A systematic review and meta-analysis. J. Med. Virol..

[4] Kellam P, Barclay W (2020). The dynamics of humoral immune responses following SARS-CoV-2 infection and the potential for reinfection. J. Gen. Virol..

[5] Yamayoshi S (2021). Antibody titers against SARS-CoV-2 decline, but do not disappear for several months. EClinicalMedicine.

[6] Li G (2020). Coronavirus infections and immune responses. J. Med. Virol..

[7] Ho MS (2005). Neutralizing antibody response and SARS severity. Emerg. Infect. Dis..

[8] Park WB (2015). Kinetics of serologic responses to MERS coronavirus infection in humans, South Korea. Emerg. Infect. Dis..

[9] Zhao J (2020). Antibody responses to SARS-CoV-2 in patients with novel coronavirus disease 2019. Clin. Infect. Dis. Off. Publ. Infect. Dis. Soc. Am..

[10] Wang Y (2020). Kinetics of viral load and antibody response in relation to COVID-19 severity. J. Clin. Investig..

[11] Liu L (2020). High neutralizing antibody titer in intensive care unit patients with COVID-19. Emerg. Microbes Infect..

[12] Post N (2020). Antibody response to SARS-CoV-2 infection in humans: A systematic review. PLoS ONE.

[13] Cevik M, Kuppalli K, Kindrachuk J, Peiris M (2020). Virology, transmission, and pathogenesis of SARS-CoV-2. BMJ.

[14] Liu R (2020). Analysis of adjunctive serological detection to nucleic acid test for severe acute respiratory syndrome coronavirus 2 (SARS-CoV-2) infection diagnosis. Int. Immunopharmacol..

[15] Busani S, Damiani E, Cavazzuti I, Donati A, Girardis M (2016). Intravenous immunoglobulin in septic shock: Review of the mechanisms of action and meta-analysis of the clinical effectiveness. Minerva Anestesiol..

[16] Bermejo-Martín JF (2014). Immunoglobulins IgG1, IgM and IgA: A synergistic team influencing survival in sepsis. J. Intern. Med..

[17] Justel M (2013). IgM levels in plasma predict outcome in severe pandemic influenza. J. Clin. Virol. Off. Publ. Pan Am. Soc. Clin. Virol..

[18] Wang Y (2021). Convalescent plasma may be a possible treatment for COVID-19: A systematic review. Int. Immunopharmacol..

[19] Joyner MJ (2021). Convalescent plasma antibody levels and the risk of death from Covid-19. N. Engl. J. Med..

[20] Tan E, Song J, Deane AM, Plummer MP (2021). Global impact of coronavirus disease 2019 infection requiring admission to the ICU: A systematic review and meta-analysis. Chest.

[21] Grasselli G (2020). Risk factors associated with mortality among patients with COVID-19 in intensive care units in Lombardy, Italy. JAMA Intern. Med..

[22] Fang X (2020). Epidemiological, comorbidity factors with severity and prognosis of COVID-19: A systematic review and meta-analysis. Aging.

[23] Flythe JE (2021). Characteristics and outcomes of individuals with pre-existing kidney disease and COVID-19 admitted to intensive care units in the United States. Am. J. Kidney Dis..

[24] Sahin U (2020). COVID-19 vaccine BNT162b1 elicits human antibody and T(H)1 T cell responses. Nature.

[25] Cascella, M., Rajnik, M., Aleem, A., Dulebohn, S. C. & Di Napoli, R. In *StatPearls* (StatPearls Publishing Copyright © 2021, StatPearls Publishing LLC., 2021).

[26] The Japan Times. *Japan Gives First COVID-19 Vaccinations to Tokyo Health Workers*. https://www.japantimes.co.jp/news/2021/02/17/national/vaccination-rollout-begins/. (Accessed 26 May 2021).

[27] National Institute of Infectious Diseases.* Current Situation of Infection, February 24, 2021*. https://www.niid.go.jp/niid/en/2019-ncov-e/10215-covid19-ab25th-en.html. (Accessed 26 May 2021).

[28] Yamakawa K (2021). Japanese rapid/living recommendations on drug management for COVID-19. Acute Med. Surg..

[29] Long QX (2020). Antibody responses to SARS-CoV-2 in patients with COVID-19. Nat. Med..

[30] Xiang F (2020). Antibody detection and dynamic characteristics in patients with coronavirus disease 2019. Clin. Infect. Dis. Off. Publ. Infect. Dis. Soc. Am..

[31] Mokobio Biotechnology R&D Center. *COVID-19 Antibody Test*. https://www.mokobious.com/copy-of-covid-19-igm-igg-test-quant (Accessed 26 April 2021).

[32] Mokobio Biotechnology R&D Center. https://www.accessdata.fda.gov/cdrh_docs/presentations/maf/maf3339-a001.pdf (Accessed 6 May 2021).

[33] Abbott.* Advancing Diagnostics of COVID-19*. https://www.corelaboratory.abbott/int/en/offerings/segments/infectious-disease/sars-cov-2. (Accessed 27 July 2021).

