# Supplementary material for: Low SARS-CoV-2 antibody titers may be associated with poor clinical outcomes for patients with severe COVID-19
Source: Sci Rep. 2022 Jun 1;12:9147. doi: 10.1038/s41598-022-12834-w (PMC9159042; doi:10.1038/s41598-022-12834-w)
Supplement: Supplementary file 1 — Supplementary Information. [file 41598_2022_12834_MOESM1_ESM.docx]

**Supplementary information**

**Low SARS-CoV-2 Antibody Titers may be Associated with Poor Clinical Outcomes for Patients with Severe COVID-19**

Mumon Takita^1^, Toru Yoshida^1^, Tomoya Tsuchida^2^, Yu Nakagama^3^, Yasutoshi Kido^3^, Shotaro Suzuki^4^, Mitsuru Imamura^4^, Kimito Kawahata^4^, Goji Shimizu^1^, Hideki Yoshida^1^, Daiki Morikawa^1^,

Takeshi Kawaguchi^1^, Shuichi Fujii^1^, Jumpei Tsukuda^1^, Takako Motohashi^5^, Shigeki Fujitani^1*^


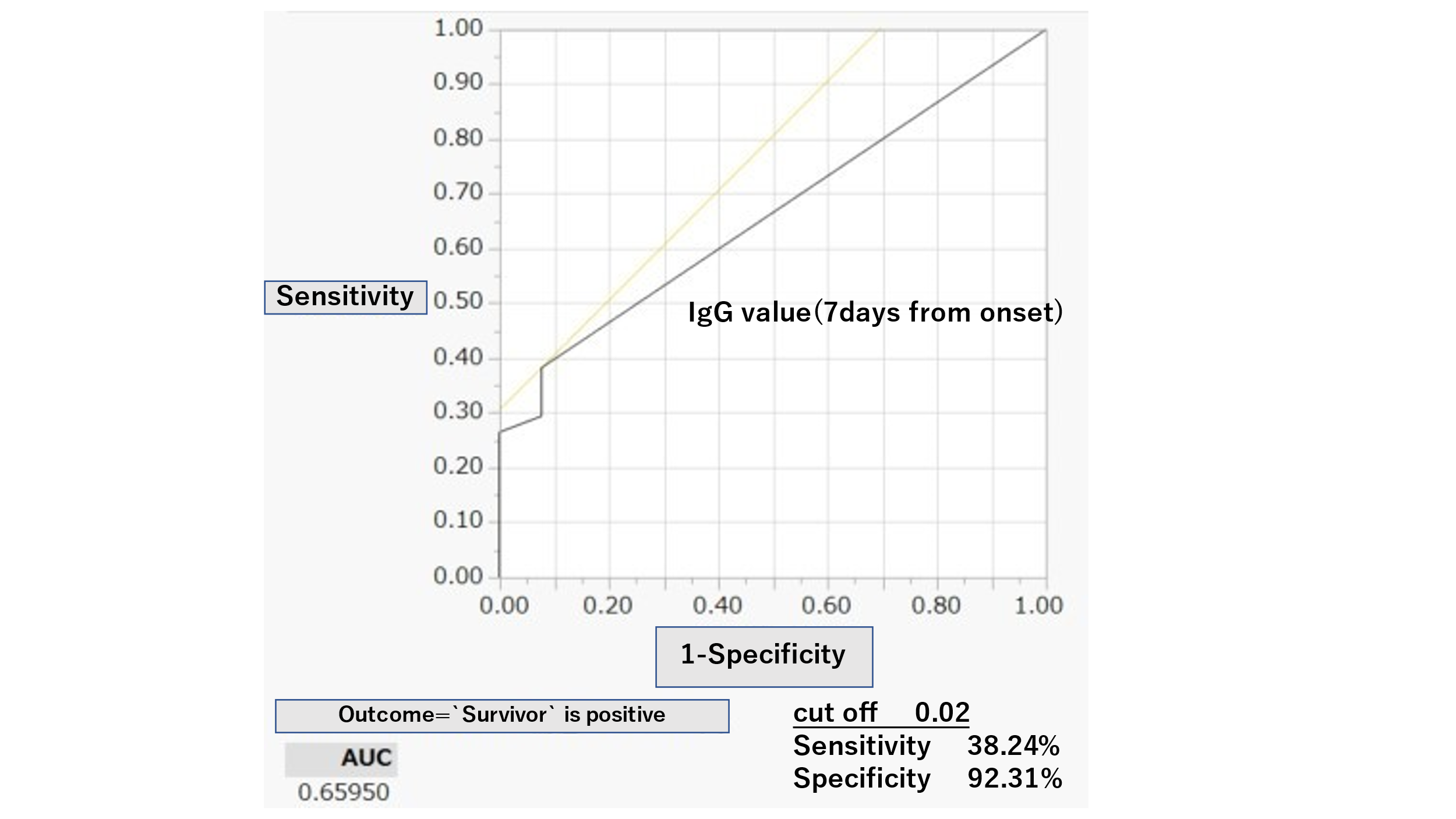


**Supplementary Figure S1.** Setting the 7-day cutoff values for IgG.

AUC, area under the concentration-time curve; IgG, immunoglobulin G


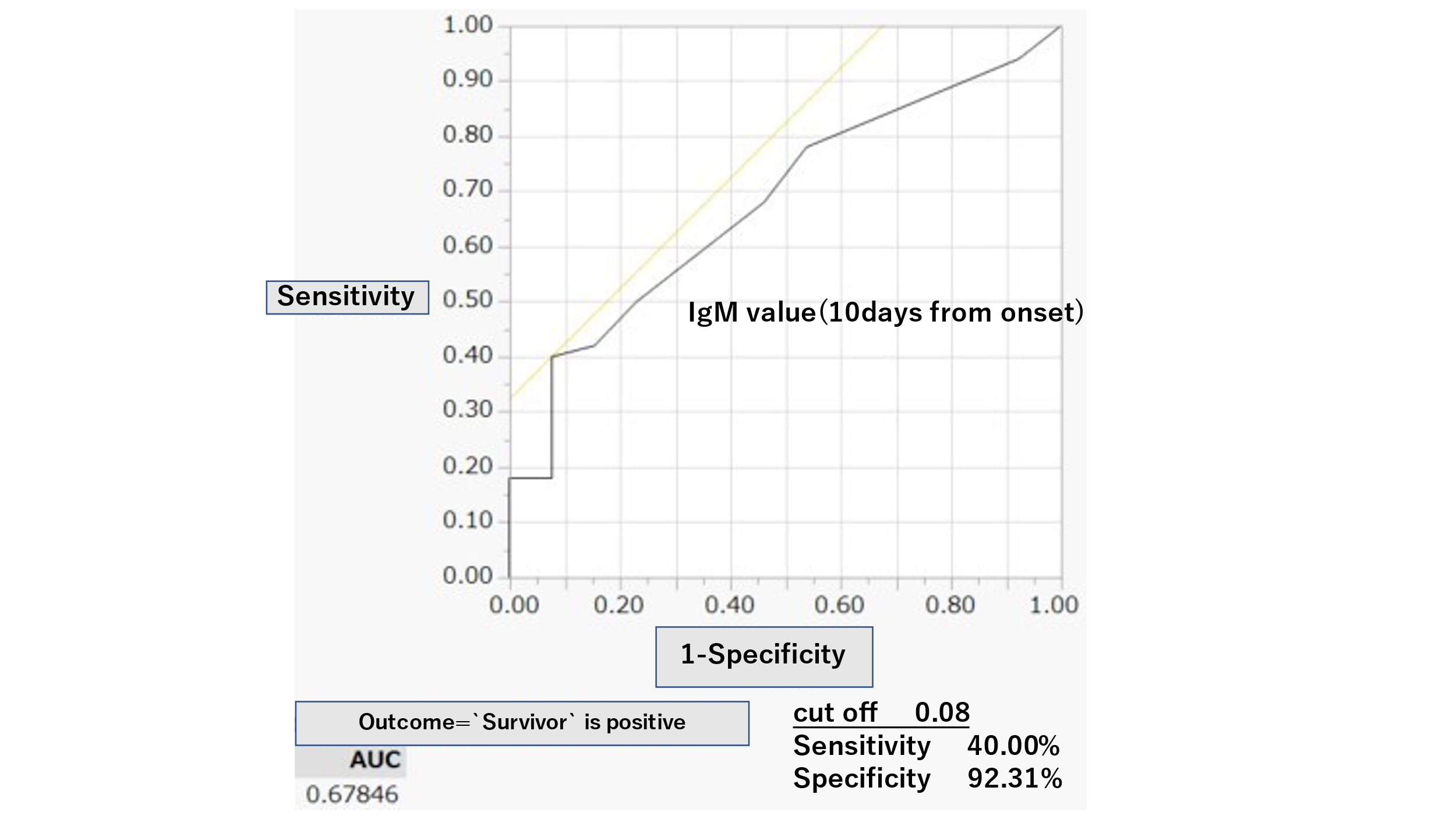


**Supplementary Figure S2.** Setting the 10-day cutoff values for IgM.

AUC, area under the concentration-time curve; IgM, immunoglobulin M


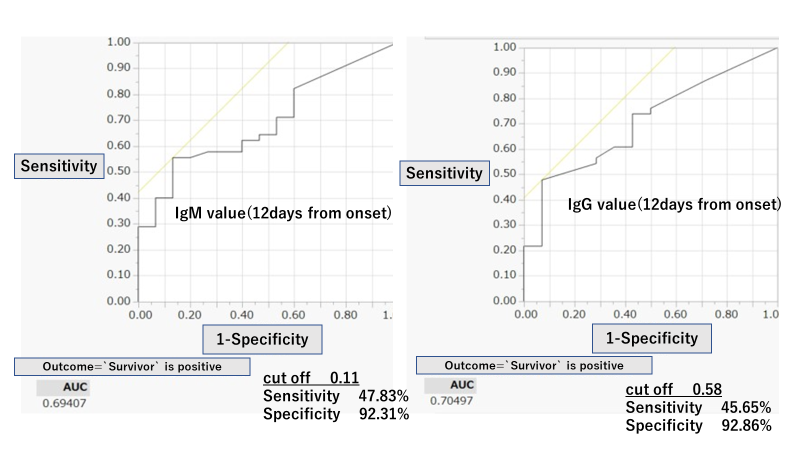


**Supplementary Figure S3.** Setting the 12-day cutoff values for IgM and IgG.

AUC, area under the concentration-time curve; IgM, immunoglobulin M; IgG, immunoglobulin G


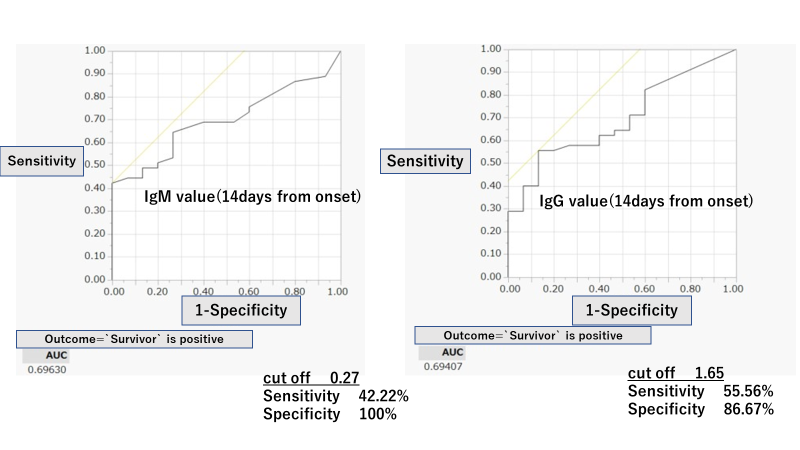


**Supplementary Figure S4.** Setting the 14-day cutoff values for IgM and IgG.

AUC, area under the concentration-time curve; IgM, immunoglobulin M; IgG, immunoglobulin G


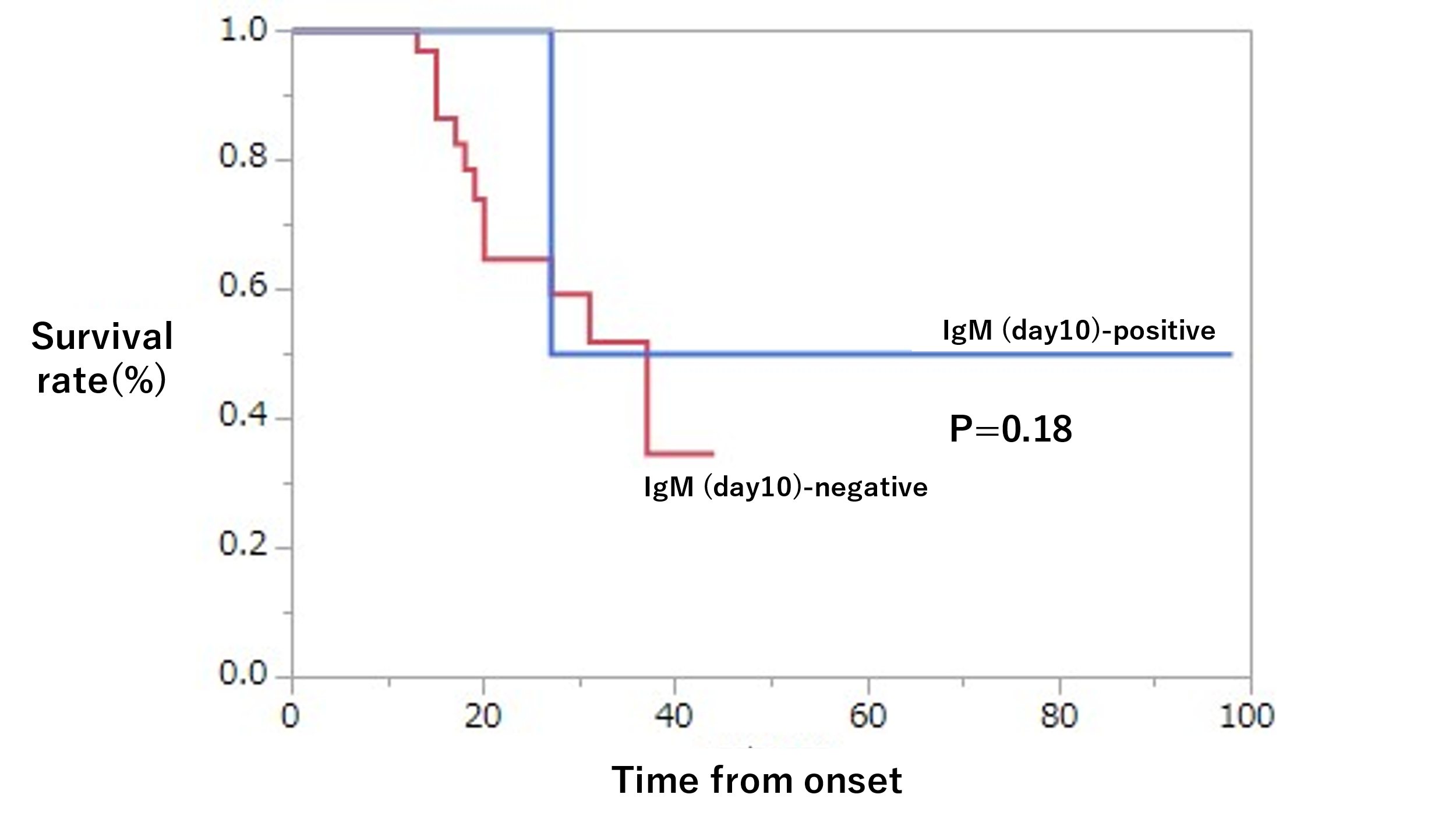


**Supplementary Figure S5.** Kaplan-Meier curve on day 10.

　　　　 IgM positive (day 10) was defined as IgM (day 10) ≧0.08.


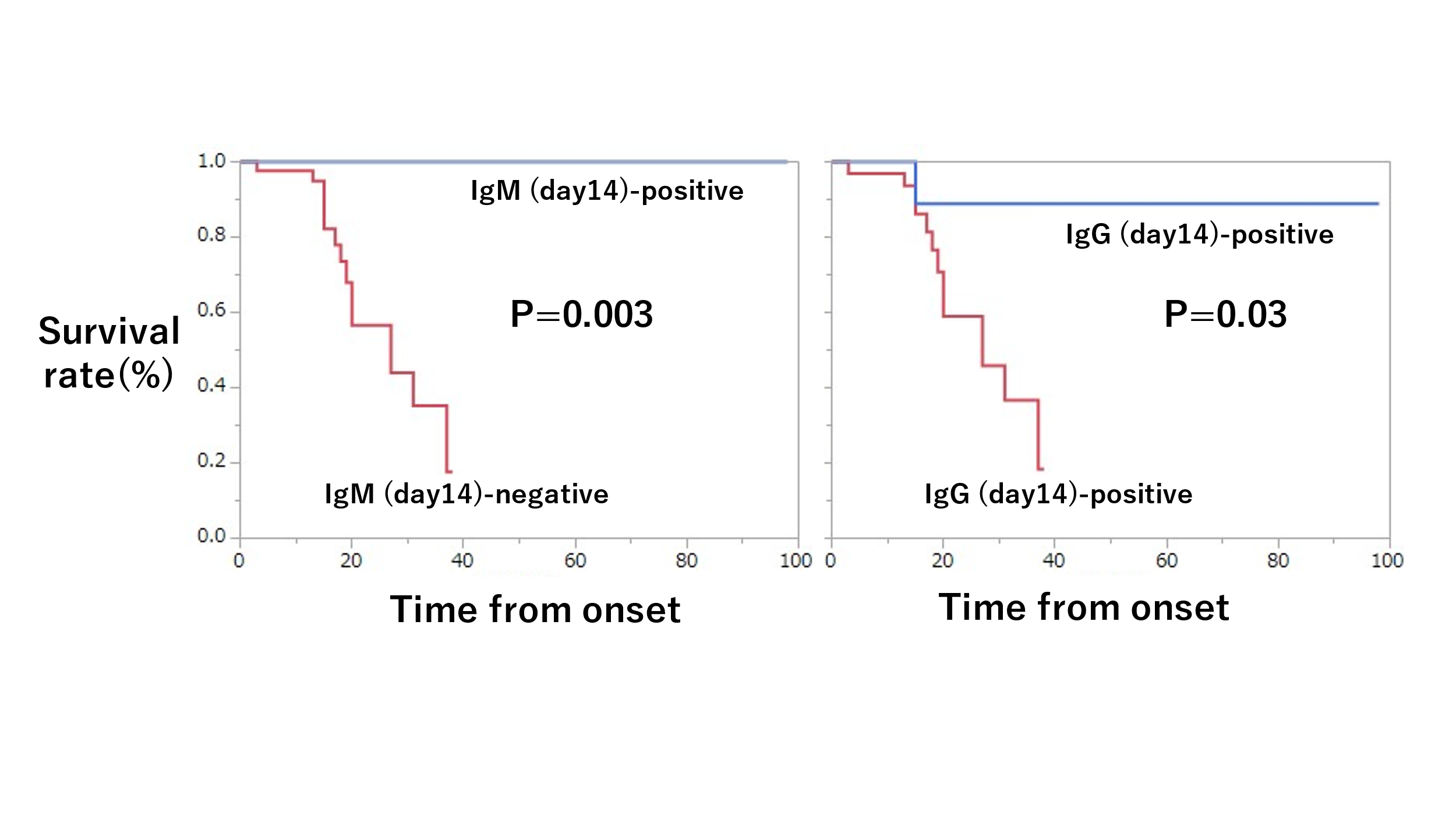


**Supplementary Figure S6.** Kaplan-Meier curve on day 14

IgM positive (day 14) was defined as IgM (day14) ≧0.27.

IgG positive (day 14) was defined as IgG (day 14) ≧1.65.

**Supplementary Table S7.** Correlation of antibody titers between Abbott and Mokobio

|  |  | **Rank correlation coefficient (Spearman)** |
| --- | --- | --- |
| **IgM** | **Abbott* vs. Mokobio**** | **0.78** |
| **IgG** | **Abbott* vs. Mokobio**** | **0.93** |

*Architect SARS-CoV-2 IgM and ARCHITECT SARS-CoV-2 IgG II Quant antibody measurement kits (Abbott, Sligo, Ireland)

** SARS-CoV-2 IgM and IgG Quantum Dot Immunoassay (Mokobio Biotechnology R&D Center, Rockville, MD, USA)

**Supplementary Table S8.** Fitting multivariate models


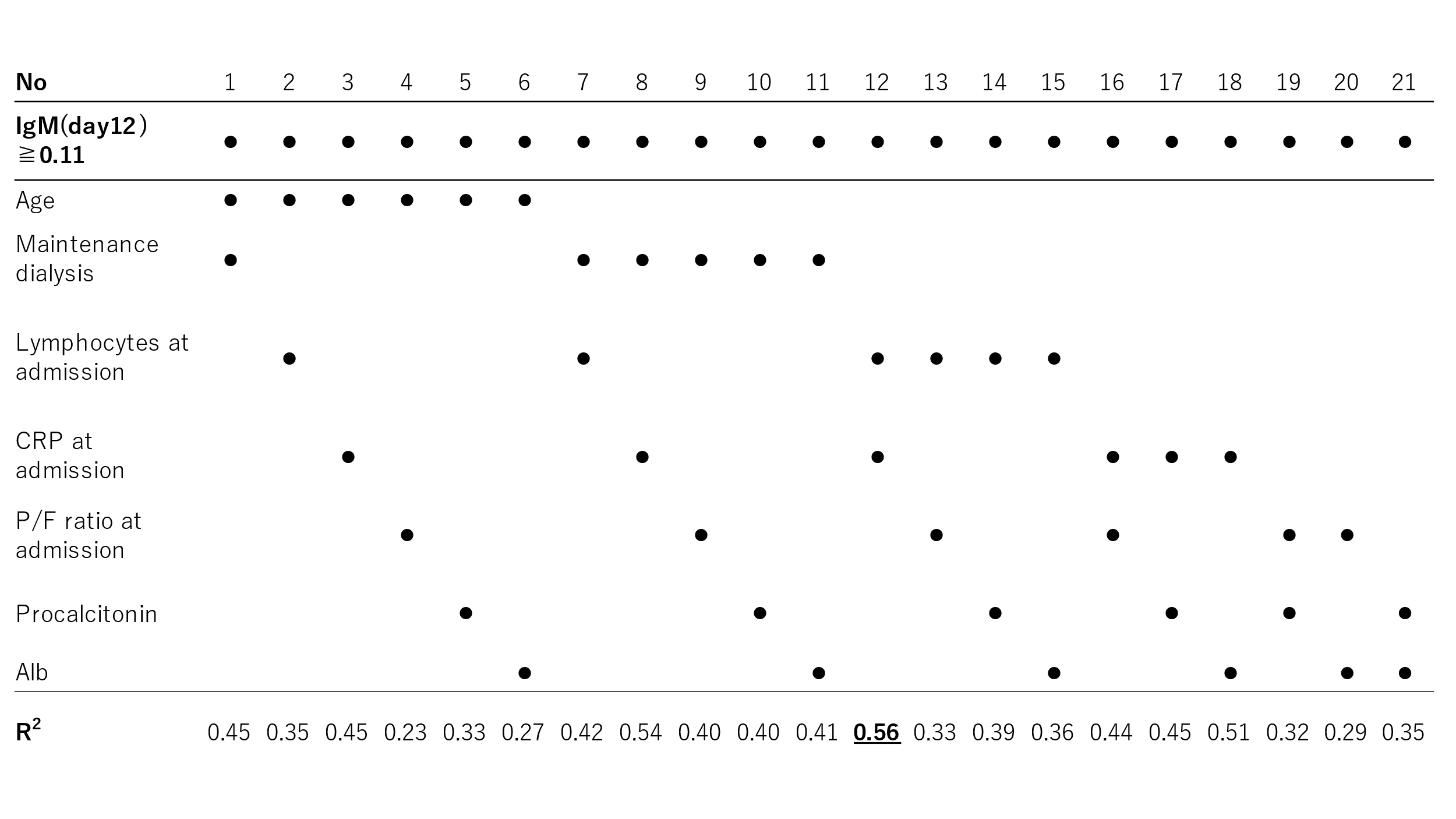


The model with the best fit (highest R^2^) was selected among the 21 models. In this table, No. 12 (Lymphocytes at admission, CRP at admission) was the best fitting model and was used to perform multivariate analysis (Table 4).

**Supplementary Table S9.** Fitting multivariate models


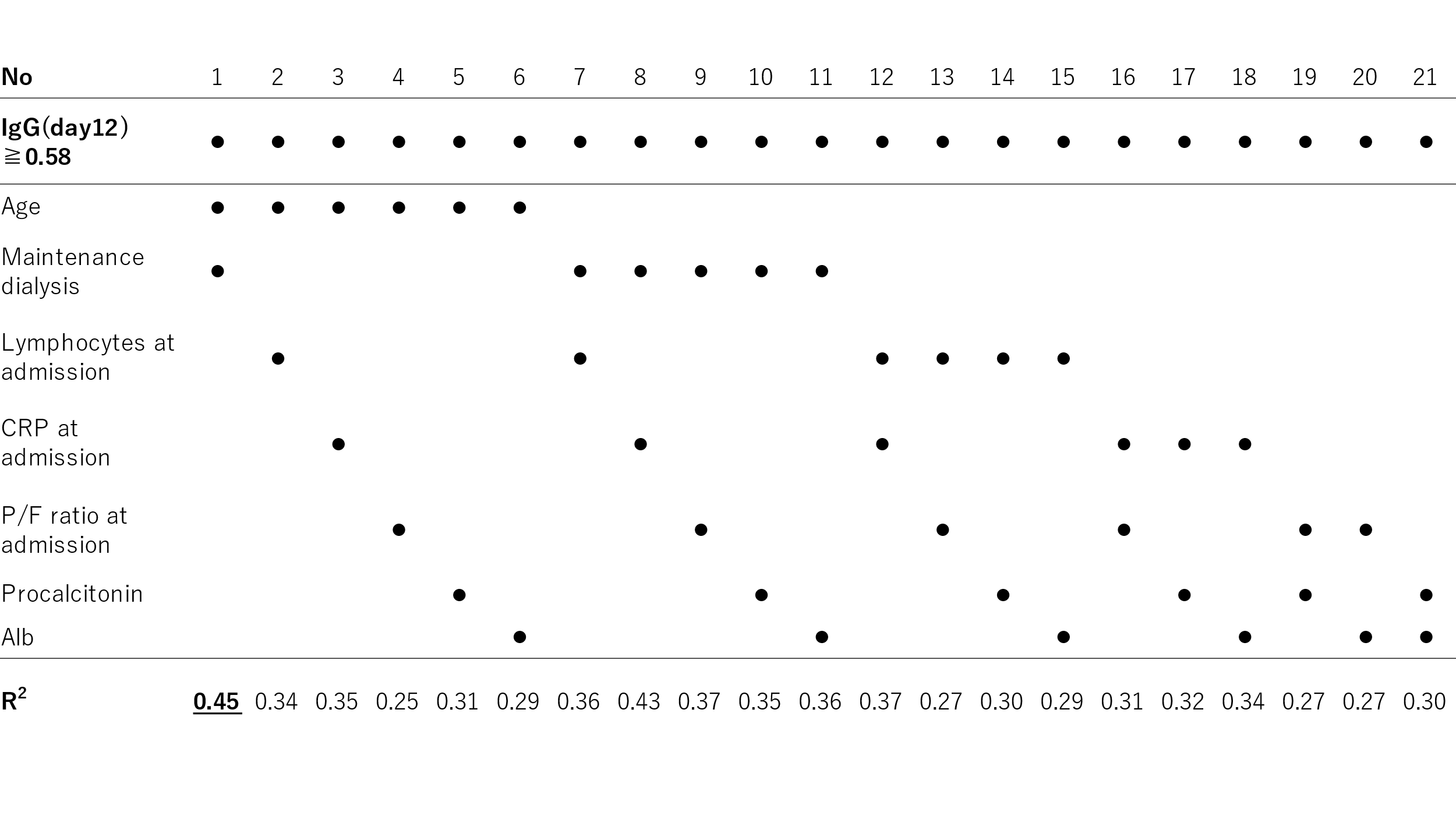


The model with the best fit (the highest R^2^) was selected among the 21 models. In this table, No. 1 (Age, Maintenance dialysis) was the best fitting model, and it was used to perform multivariate analysis (Table 4).

**Supplementary Table S10.** Fitting multivariate models


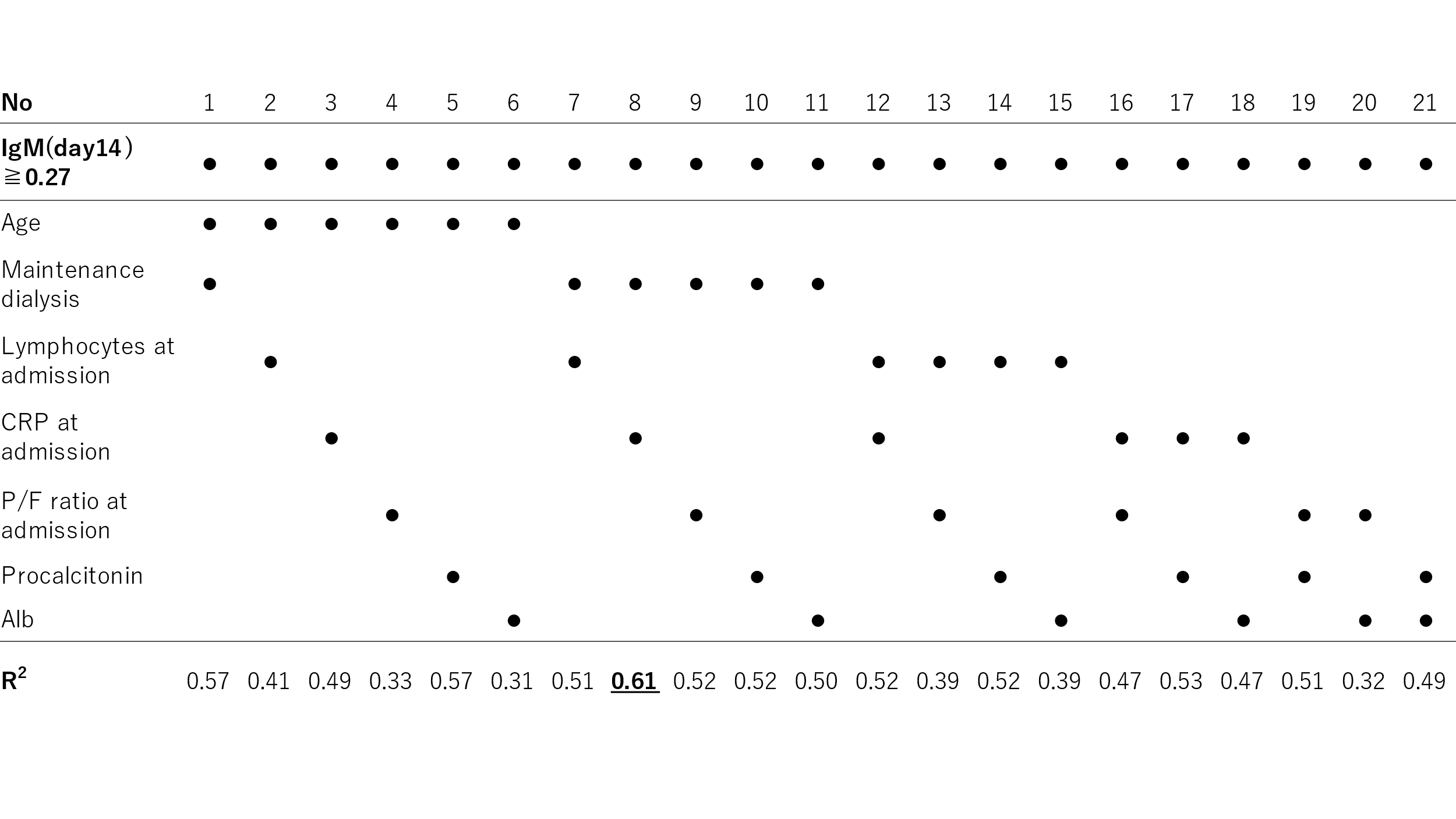


The model with the best fit (the highest R^2^) was selected among the 21 models. In this table, No. 8 (Maintenance dialysis, CRP at admission) was the best fitting model and was used to perform multivariate analysis (Table 4).

**Supplementary Table S11.** Fitting multivariate models


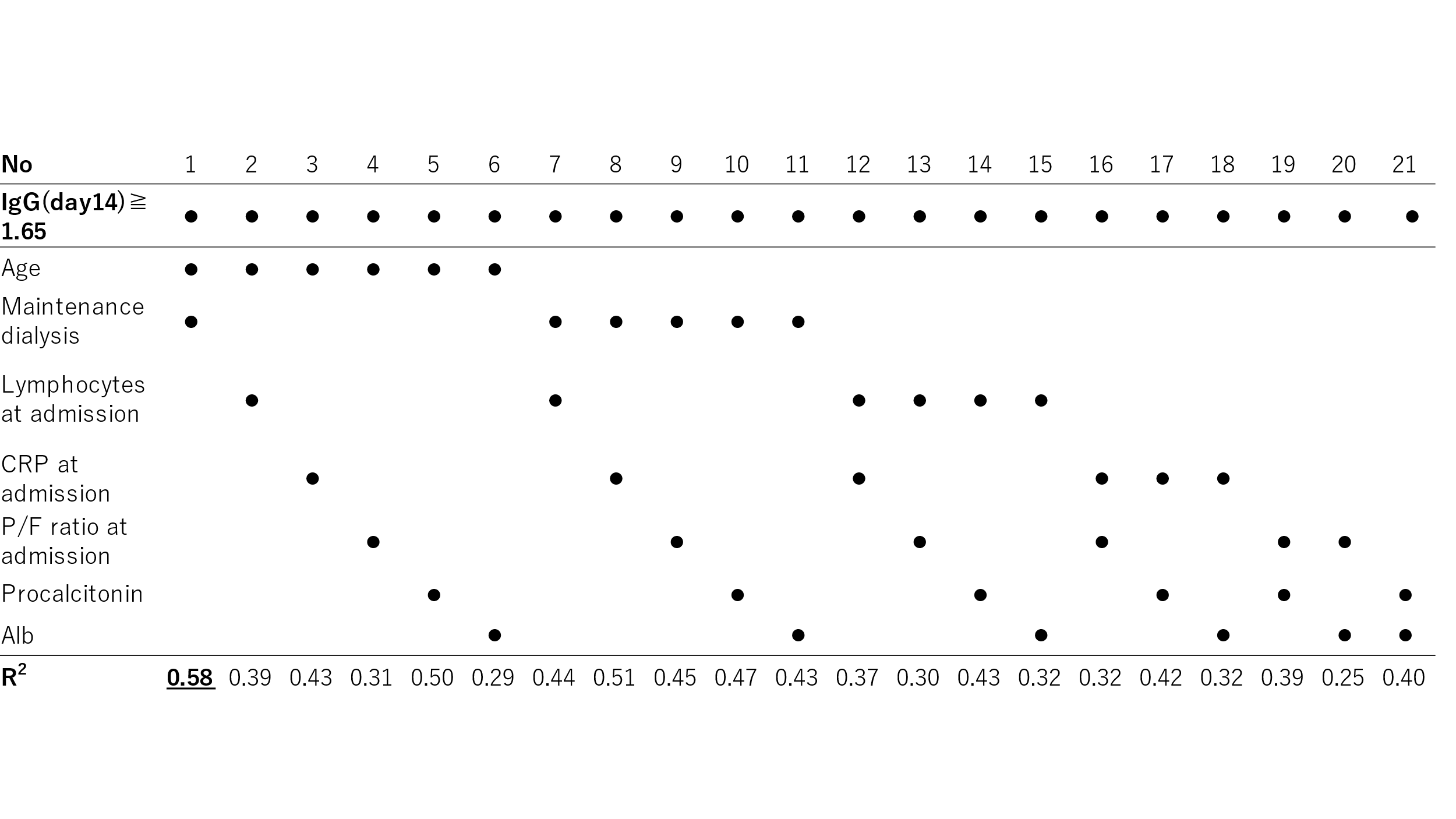


The model with the best fit (the highest R^2^) was selected among the 21 models. In this table, No. 1 (Age, Maintenance dialysis) was the best fitting model and was used to perform multivariate analysis (Table 4).
